# Research on Plant Landscape Design of Urban Industrial Site Green Space Based on Green Infrastructure Concept

**DOI:** 10.3390/plants14050747

**Published:** 2025-03-01

**Authors:** Jiahui Ai, Myun Kim

**Affiliations:** Department of Industrial Design, Pukyong National University, Busan 48513, Republic of Korea; aijiahui007@gmail.com

**Keywords:** industrial sites, green infrastructure, plant landscape design, heat island effect, microclimate simulation

## Abstract

With the acceleration of the global urbanization process, more and more industrial plants are being abandoned, which puts great pressure on urban ecology and land resource management. These abandoned industrial spaces not only lead to persistent pollution problems, but also exacerbate the urban heat island effect, leading to a worsening microclimate. To address these issues, the concept of green infrastructure (GI) has emerged as a sustainable ecological restoration strategy, and it is an important tool for urban renewal and industrial land transformation. In this study, the landscape environment of the industrial site of Henrichshutte in Germany and the surrounding industrial plant was taken as an example, and ecological restoration and plant landscape design were carried out using the GI concept. Two climate simulation tools, ENVI-met and WindPerfect DX, were comprehensively adopted to simulate the environment of the site in detail. Based on an analysis of the potential temperature, PMV, wind speed, and UTCI data of the site, it was demonstrated that the plant landscape improved the microclimate of the industrial plant. The results show that the reasonable allocation of plants can effectively reduce surface temperature and building temperature, increase air humidity, alleviate the local heat island effect, and enhance the thermal comfort of the human body. The simulation results highlight the practical application value of the GI concept in improving the ecological benefit, social function, and landscape aesthetics of industrial land. This study provides a new idea for the ecological restoration and environmental optimization of urban industrial land through the combination of green infrastructure and plant landscape design, and emphasizes the important role of green infrastructure in alleviating the urban heat island effect and promoting the sustainable development of urban landscape spaces.

## 1. Introduction

The number of industrial sites in modern cities is gradually increasing. These abandoned sites, due to the cessation of industrial activities, become “grey spaces” in the urban environment. Not only are they not suitable for continuous industrial production, but the severe pollution and dilapidated landscape also hinder their direct transformation into livable spaces. This leads to the wasting of urban land resources, and causes serious pollution to the urban groundwater, air, and soil environment [1]. Urban industrial sites refer to abandoned or abandoned industrial land, buildings and facilities located in or on the outskirts of a city. These include factories, warehouses, railway facilities, power stations, mining areas, and other industrial structures or areas. The formation of these sites is often driven by urbanization, industrial restructuring, stricter environmental regulations, relocation of production facilities, or plant closures. Once abandoned, these areas face problems such as pollution, waste accumulation and under-utilization of resources. They form a “grey zone” inside the city and have a negative impact on the aesthetics and ecological functions of the city [2].

The emergence of urban industrial sites stems from several factors. First, rapid urbanization has increased spatial demands in cities. Industrial areas that were originally located on city peripheries have gradually been surrounded by residential and commercial zones. Consequently, these industrial plants are often relocated due to land shortages, leaving behind industrial sites [3]. Additionally, traditional manufacturing and heavy industries are increasingly being replaced by new industries and high-tech sectors. This economic transformation has resulted in the closure or relocation of numerous traditional industrial plants, leaving industrial land abandoned [4]. Furthermore, some industrial facilities fail to meet modern environmental standards and are forced to shut down or relocate, leaving behind contaminated land that becomes industrial sites. These areas often retain hazardous materials that pollute soil and water [5]. In industrial sites, extensive exposed hard surfaces and abandoned building materials absorb solar heat, creating localized high-temperature zones. Additionally, the lack of vegetation cover in industrial land significantly intensifies the urban heat island effect. It also leads to an unfavorable wind environment on the site, which not only reduces its usability but also negatively impacts the thermal comfort of the surrounding area [6].

However, industrial sites hold significant potential for reuse. Through proper renovation and optimized design, these areas can be transformed into urban green spaces, parks or ecological restoration zones, seamlessly reintegrating into the urban functional framework. Guided by the concept of green infrastructure, plant landscapes can play a key role in restoring and repurposing urban industrial sites [7]. Through proper planning and design, these areas can be transformed into new drivers of urban development, making their reuse a key theme in urban planning. In recent years, the concept of green infrastructure (GI) has gained significant attention, emerging as a key ecological solution in urban planning [8].

Green infrastructure (GI) is a holistic urban and regional planning concept centered on a network of interconnected green spaces. These spaces include urban greenways, parks, wetlands, rain gardens, and native vegetation. The core principle of GI is to harness natural elements to create comfortable and functional environments. Unlike traditional gray infrastructure, which relies on hard pavements and concrete, GI incorporates features such as native plants, green roofs, and water elements to enhance urban design. It plays a crucial role in promoting ecological sustainability and improving urban livability [9,10,11].

The integration of green infrastructure in urban areas provides numerous benefits. From a climate regulation perspective, it effectively reduces thermal radiation, absorbs heat emissions from surrounding areas, and enhances overall comfort. In terms of wind environment improvement, it helps moderate wind speed, reduce evaporation, and create more comfortable environmental conditions. Additionally, green infrastructure plays a vital role in air purification by capturing particulate pollutants and improving air quality [12,13]. Several successful international cases have demonstrated the role of green infrastructure in revitalizing industrial sites. For example, Nordstern Park in Germany and Millennium Park in Chicago, USA have successfully transformed former industrial areas through ecological restoration and landscape redevelopment. These projects have not only improved environmental conditions but also provided valuable cultural and recreational spaces for urban residents.

These cases provide strong theoretical support for this study. Within the framework of green infrastructure, the primary evaluation indicators used in this research include potential temperature, predicted mean vote (PMV), wind speed, and universal thermal climate index (UTCI). PMV and UTCI remain the most widely used objective indicators for assessing thermal comfort. This method is applicable not only to the evaluation of thermal comfort in existing buildings but also to the simulation and prediction of thermal comfort in outdoor environments [14,15].

By integrating ENVI-met and WindPerfect DX for simulations, data on potential temperature, PMV, wind velocity, and UTCI were obtained to analyze the role of vegetation in regulating the microclimate. These simulation results provide a scientific basis for optimizing the landscape design of industrial brownfields [16,17,18,19]. The aim of this study was to explore how green infrastructure can be applied to the ecological restoration and landscape design of industrial sites. Specifically, the focus of this research is on the rational configuration of plant landscapes to enhance the ecological benefits of urban industrial land. By modeling the outdoor environment of the industrial site and designing a plant-based landscape, climate-related data were simulated. The findings effectively validate the role of plant design in improving environmental temperature, wind conditions, and thermal comfort.

## 2. Materials and Methods

### 2.1. Choice of Study Site

The Henrichshütte industrial site, located in Hattingen, Germany, was selected for this study. As a traditional steel plant in the Ruhr region, it had been in operation since the late 19th century. Decades of industrial activity resulted in severe environmental pollution, particularly heavy metal contamination, as well as wastewater and air emissions, which significantly impacted the surrounding ecosystem [20].

After the facility was abandoned, the site gradually became dominated by exposed hard surfaces and extensive gray pavement, exacerbating the urban heat island effect. This issue is particularly pronounced in summer when concrete and metal structures absorb solar radiation, leading to a significant rise in local temperatures and reduced thermal comfort. Therefore, this study focused on exploring how the integration of green infrastructure and thoughtful plant landscape design can help improve the site’s microclimate and ecological conditions.

### 2.2. Plant Configuration of the Study Site

Based on the concept of green infrastructure, a planting design was implemented at the study site. Planting design is a form of landscape design that integrates plant elements with the environment and spatial composition [21]. To examine the impact of plant landscapes on the microclimate of industrial sites, a multi-layered planting approach was adopted, utilizing native plant species as the foundation to establish a diverse and structured plant community.

The planting design consisted of three layers: the tree layer, shrub layer, and ground cover layer. For the tree layer, tall tree species such as European ash, European beech, Norway maple, and Ginkgo were selected to enhance shading and improve the site’s thermal environment.

For the shrub layer, species such as Spiraea, Elderberry, and Lilac were selected. These shrubs not only play a significant role in landscape beautification but also contribute to microclimate regulation by providing additional shading with their dense foliage, facilitating air circulation, and helping maintain local humidity levels.

For the ground cover layer, plants such as Violet and Cornflower were chosen. These ground covers help control surface temperature, retain soil moisture, and reduce water evaporation, further mitigating the surface heat radiation effect [22].

In the plant landscape design, careful consideration was given to the ecological functions and aesthetic value of each vegetation layer. By integrating the site’s functional requirements, the overall design not only supports ecological restoration but also enhances visitor comfort and site usability.

### 2.3. ENVI-Met and WindPerfect DX Simulation

In the measurement and simulation of microclimates and thermal comfort in spatial environments, ENVI-met5.7.1 is a commonly used simulation tool [23]. For wind environment simulation, WindPerfect DX demonstrates better performance, as it can model air circulation around building environments to assist in the rational planning and layout of buildings and landscape plants [24]. To conduct a comprehensive evaluation of the spatial environment and investigate the impact of green infrastructure on comfort and wind environment, both ENVI-met and WindPerfect DX simulation tools were combined to perform data simulations for the Henrichshütte steel plant and the surrounding factory site area.

(1) Site Model Development

A three-dimensional spatial model of the Henrichshütte steel plant and its surrounding environment was constructed using modeling software. Additionally, a control group was established, consisting of two conditions: one representing the original green space without plant design, and the other representing an ecological green space with plant design. The setup of the control groups allows for a direct comparison to demonstrate the impact of plant design on the spatial microclimate and human comfort.

(2) ENVI-met Simulation Data

Potential Temperature: This is a meteorological index used to describe air quality. It represents the temperature that air would reach if it were to undergo an adiabatic process (such as rising or descending) without exchanging heat with the surrounding environment [25].

PMV: The predicted mean vote (PMV) is an index that predicts the overall thermal comfort of the human body by considering factors such as temperature, humidity, wind speed, and other environmental variables. It provides a comprehensive evaluation of how different microclimatic conditions affect human comfort [26].

(3) WindPerfect DX Simulation Data

Wind Speed: Wind speed data is crucial for understanding the intensity of wind flow in specific areas. It plays a vital role in evaluating air circulation, as well as the cooling effect of wind in the environment [27].

UTCI: The universal thermal climate index (UTCI) is used to assess thermal comfort in outdoor environments. It provides an intuitive measure of how the human body perceives heat in a given spatial environment, taking into account factors such as air temperature, humidity, wind speed, and solar radiation [28].

Through the analysis of the above data, the microclimatic changes of the simulated site at different time points were examined. This allowed for a deeper analysis of the role that plant landscapes in green infrastructure play in reducing site temperature and enhancing human thermal comfort.

### 2.4. Data Analysis

After completing the site simulation calculations, a comparative analysis was conducted on the site temperature, PMV, wind speed, and UTCI data. By comparing these data, this study explores the role of plant design in improving the microclimate [28].

## 3. Relevant Case Analysis

Currently, numerous domestic and international cases have demonstrated the successful improvement of harsh industrial site environments through plant design. In most of these cases, the focus is on reconstructing the park’s ecosystem by integrating green infrastructure solutions. By drawing on the lessons from these exemplary cases, valuable references can be provided for the transformation and revitalization of similar industrial sites.

### 3.1. Nordstern Park

**Figure 1:**
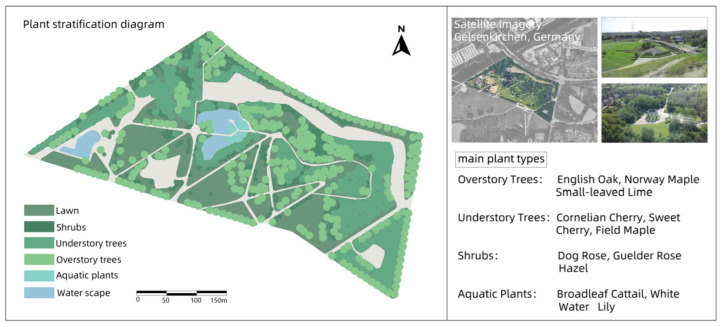
Plant landscape analysis of Nordstern Park.

Nordstern Park is a large green park located in Gelsenkirchen, Germany. Through landscape restoration and functional reconstruction, it was transformed from an old factory into a green open park. The park has large areas of green space, rivers, and modern buildings [29]. The park’s design focuses on ecological balance, combining natural landscapes with human activities to protect local animal and plant ecosystems (Figure 1).

### 3.2. Westerpark

**Figure 2:**
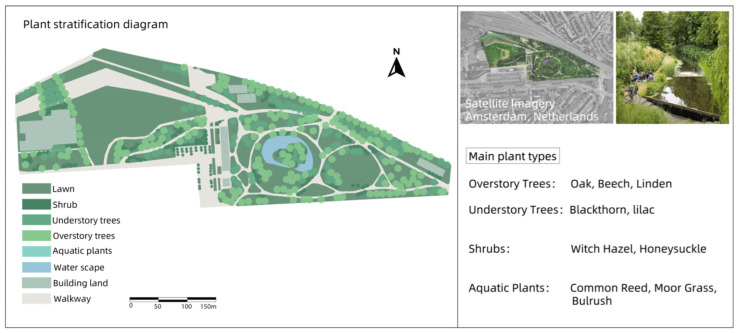
Plant landscape analysis of Westerpark.

Westerpark is a large urban park in the west of Amsterdam, the Netherlands. It is not only an ideal place for citizens and visitors to relax and enjoy nature, but also an integrated area of history, culture, and entertainment. Westerpark has a rich historical background and was once an industrial area. After restoration, this area has become a modern leisure park and cultural center, becoming one of the models of urban renewal in Amsterdam. Westerpark is designed to combine natural and urban elements, with large areas of green space, trees, small lakes, and walking paths, perfect for walking, running, and biking (Figure 2).

### 3.3. Millennium Park

**Figure 3:**
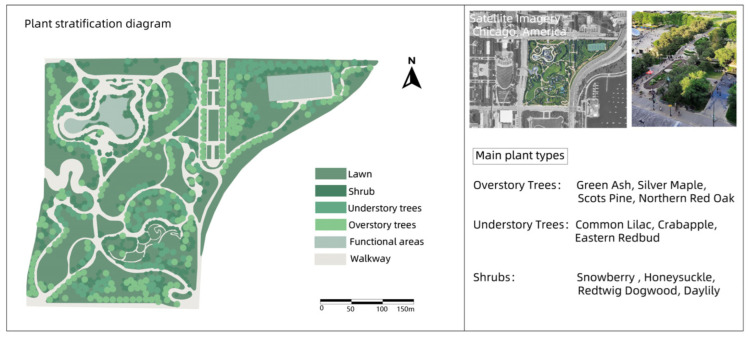
Plant landscape analysis of Millennium Park.

Built on top of an old railroad and parking lot, this park is part of a plan to renew downtown Chicago and transform an industrial area into a recreational space for citizens and visitors. The plant design of Millennium Park, especially in the Lurie Garden, embodies a unique design philosophy and ecological sustainability. Lurie Garden is a core botanical landscape area of the park, covering approximately five acres, and its plant design incorporates native plants, ecological restoration concepts, and symbolic historical and cultural elements. Lurie Garden makes extensive use of native North American plants, such as tall grass prairie plants and wildflowers, which are important in the local ecology, and not only beautify the environment, but also support native wildlife communities such as bees, butterflies, and birds [30]. By selecting plants that bloom and bear fruit in different seasons, the garden ensures that the landscape changes throughout the seasons. Tulips in spring, prairie flowers in summer, grasses in autumn, and hardy vegetation in winter all combine to create an ever-changing visual feast. The plants in the garden are designed with a focus on sustainability, and the plants chosen are mostly adapted to local climatic conditions, reducing the need for water, fertilizers, and chemicals. This not only reduces maintenance costs, but also reduces environmental impact (Figure 3).

### 3.4. Buttes–Chaumont Park

Buttes–Chaumont Park is a large public park in the 19th arrondissement of Paris, France. Buttes–Chaumont Park was formerly an abandoned limestone quarry. The area was a barren, desolate piece of land in the 19th century, used for quarrying, landfills, and public executions. In the 1860s, Georges-Eugene Haussmann, an urban planner under Napoleon III, set about transforming this industrial wasteland into a beautiful public park. The park was officially opened in 1867 during the Paris Universal Exposition.

**Figure 4:**
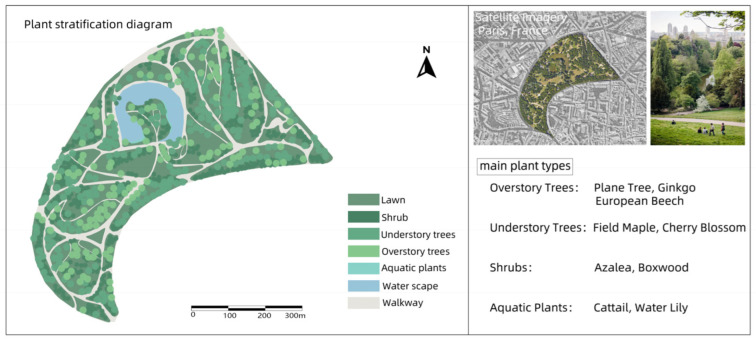
Plant landscape analysis of Buttes–Chaumont Park.

Buttes–Chaumont Park is planted with a large number of different trees and plants, including native and exotic French species such as cedars, maples, willows, and chestnuts. The plant design of the park allows for different landscape changes throughout the year. Due to its naturalized design and large areas of green space, the park is also a habitat for much wildlife, especially a variety of birds. Lakes and green spaces provide an ideal environment for animals [31] (Figure 4).

In these four cases, the core concept of green infrastructure was fully applied, especially in the ecological restoration process of industrial sites. Whether it is Nordstern Park in Germany or Westerpark in the Netherlands, these renovations have successfully restored the ecological function of these areas by introducing a large number of vegetation, water restoration, and ecological corridors. Industrial sites often have pollution problems (such as soil and water pollution), and through ecological design, these parks effectively reduce the impact of pollution on the environment and enhance biodiversity.

All four parks have improved ecosystems through the introduction of multiple layers of vegetation (trees, shrubs, ground cover), reduced the heat island effect, and increased air humidity through plant transpiration. In terms of ecological restoration, these parks pay special attention to water restoration and rainwater management, using wetlands, rain gardens, and ecological corridors to enhance the recycling and management of water resources.

Different parks have selected different plant species according to their original ecological conditions and climatic environment. For example, Nordstern Park in Germany relies more on native plants, while Millennium Park in Chicago combines native plants with ornamental flowers, reflecting the differences in plant selection between different regions. Westerpark is not only a leisure park, but also a cultural center and space for artistic activities, making full use of the architectural remains of the former gas works. Buttes–Chaumont Park puts more emphasis on the ornamental nature of the landscape, and enhances the visual appeal of the park through the shaping of the terrain and the design of cliff caves.

Through the comparative analysis of four industrial site transformation parks, the extensive application and flexibility of green infrastructure in ecological restoration and social function enhancement are demonstrated, which provides important theoretical support and basis for the application of the green infrastructure concept in the design of Henrichshutte industrial site.

## 4. Empirical Analysis

### 4.1. Henrichshutte Industrial Site

**Figure 5:**
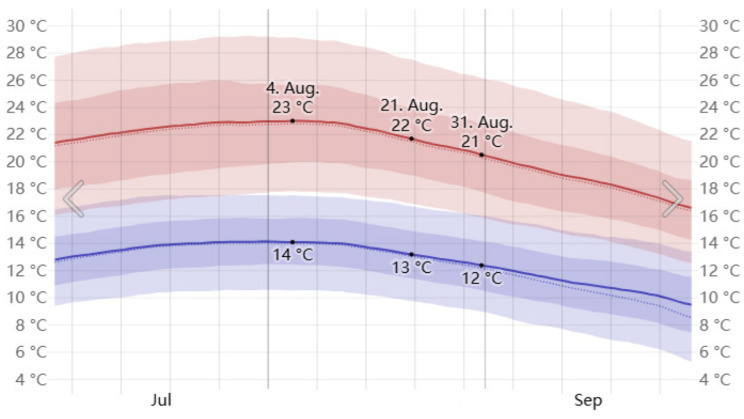
Average high and low temperatures in August in Hattingen.

**Figure 6:**
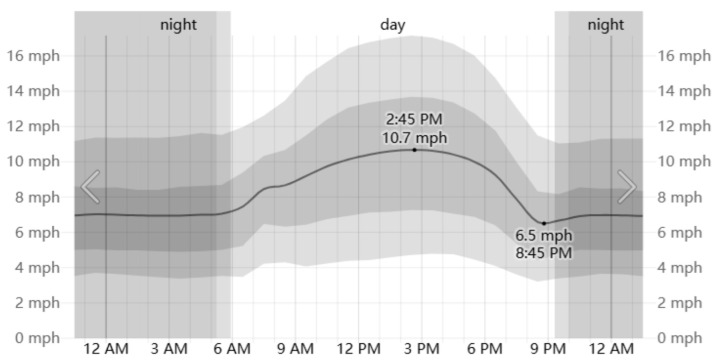
Wind speed on August 1 in Hattingen.

**Figure 7:**
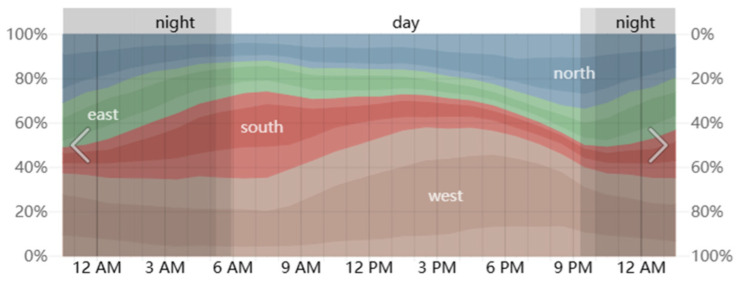
Wind direction on August 1 in Hattingen.

The Henrichshütte steel plant, located in Hattingen, Germany, is situated at 51°24′ N, 7°11′ E. Hattingen has a temperate maritime climate, with relatively mild average temperatures in the summer, though high-temperature weather can still occur. According to historical meteorological data provided by the Weather Spark platform, we analyzed the weather data for 2022, 2023, and 2024 for Hattingen and calculated the average values. The highest temperatures in Hattingen typically occur in early August. Therefore, August 1st was selected as the study period for this simulation (Figure 5, Figure 6 and Figure 7).

Henrichshütte was established in 1854 and is one of the most traditional steel mills in the Ruhr region, renowned for its production of stainless steel. However, due to frequent flooding of the Ruhr River, the area became difficult for navigation. Additionally, since 1870, the iron ore reserves were no longer sufficient to meet the needs of the smelting plant, leading to a gradual decline in its industrial prominence. Today, the Henrichshütte industrial site has been transformed into an industrial heritage museum. However, the long-term impact of industrial production has severely damaged the landscape and space, leaving it in a state of significant degradation [32]. To ensure more accurate simulation results and better reflect air flow data, we selected a simulation area of approximately 450,000 square meters, which includes both the steel plant and the newly constructed industrial buildings surrounding it.

**Figure 8:**
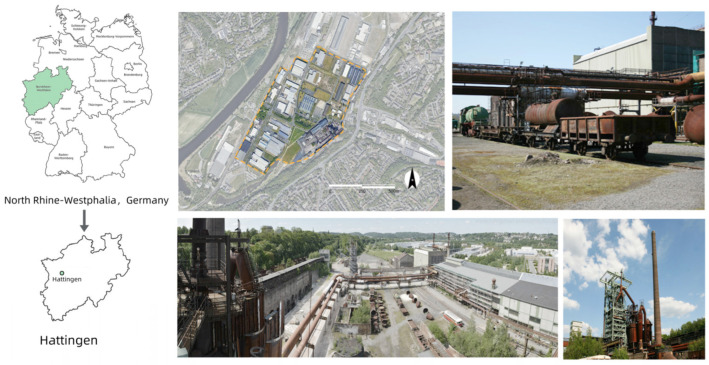
Location and current status of Henrichshutte.

Within the industrial site, we observed large areas of exposed hard surfaces and construction materials, such as metal and concrete. These environmental conditions cause the space to absorb and store significant amounts of solar radiation heat during the summer, leading to elevated local temperatures and the formation of the urban heat island effect. Additionally, the vegetation coverage around the buildings is relatively low, which hinders the creation of a favorable wind environment and reduces the overall comfort of the space (Figure 8).

The reconstruction of industrial sites is an important part of urban renewal, which not only means the functional reconstruction of abandoned sites, but also needs to pay attention to the improvement of environmental quality [33]. As an ecological transformation method, plant landscape design can restore the ecology of industrial sites and create public spaces with both cultural and social functions. Therefore, the concept of green infrastructure is introduced in this study. Green infrastructure emphasizes the use of natural ecosystems, such as plants, to enhance the environmental adaptability of spatial areas and promote sustainable development. As a part of green infrastructure, plant landscape design can effectively regulate the microclimate of the space environment and reduce the heat island effect.

In order to further understand the microclimate of the Henrichshutte industrial site and its surrounding environment, the ENVI-met5.7.1 was used to conduct a detailed temperature simulation of the area. The effect of green infrastructure design on microclimate improvement was validated through ENVI-met simulations. In the absence of any vegetation intervention, this study obtained a preliminary temperature distribution around the building. These simulation results provide baseline data for subsequent plant landscape interventions through the green infrastructure concept in order to compare environmental changes after plant configuration. The modeling capabilities of ENVI-met allow us to visually observe the temperature increases caused by buildings and hard surfaces, and use these data as a baseline value before the plant landscape intervention, and then compare the environmental changes after the plant configuration [34].

### 4.2. Site Temperature and Human Comfort Data Analysis

#### 4.2.1. Site Temperature Analysis

In the absence of plant design in the site, ENVI-met simulation results show that the temperature in the Henrichshutte industrial site buildings and surrounding areas is significantly higher than that in the nearby green spaces.

High-temperature areas: These are mainly concentrated on the southwest side of the buildings and the surrounding open areas that lack shading facilities. Due to the absence of tree cover in these areas, solar radiation directly strikes the ground, causing a significant increase in surface temperature.

Significant urban heat island effect: The building materials in the industrial site, such as steel and concrete, have a high thermal capacity and can absorb and release large amounts of heat. This temperature difference indicates that the area will become a localized heat island in the summer.

(1) Time: 00:00–10:00

At 00:00, the temperature across the entire area is relatively low, typically ranging between 20–22 °C. During this period, the temperature remains relatively uniform, as the building surfaces maintain lower temperatures due to the absence of direct solar radiation.

As time progresses to 06:00, the temperature gradually rises, especially around the buildings and open areas. Surface temperatures begin to noticeably increase, reaching between 25 and 28 °C.

**Figure 9:**
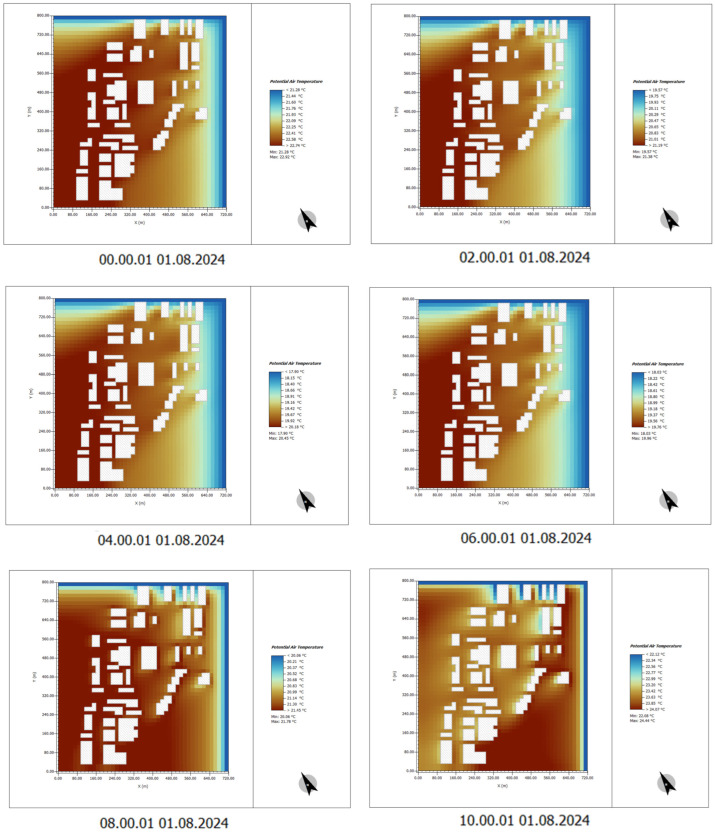
Potential temperature simulation results. Time: 0:00–10:00. (The white shape represents the building).

By 10:00, temperatures continue to rise, particularly on building surfaces, indicating that these areas have absorbed a significant amount of solar radiation, leading to a marked increase in warmth (Figure 9).

(2) Time: 12:00–22:00

By 12:00, the temperature increases significantly, with building surface temperatures reaching over 30 °C. Temperatures in open areas also rise considerably. The urban heat island effect becomes more pronounced at this time, especially in areas with buildings and exposed ground, where temperatures are much higher than in surrounding regions.

**Figure 10:**
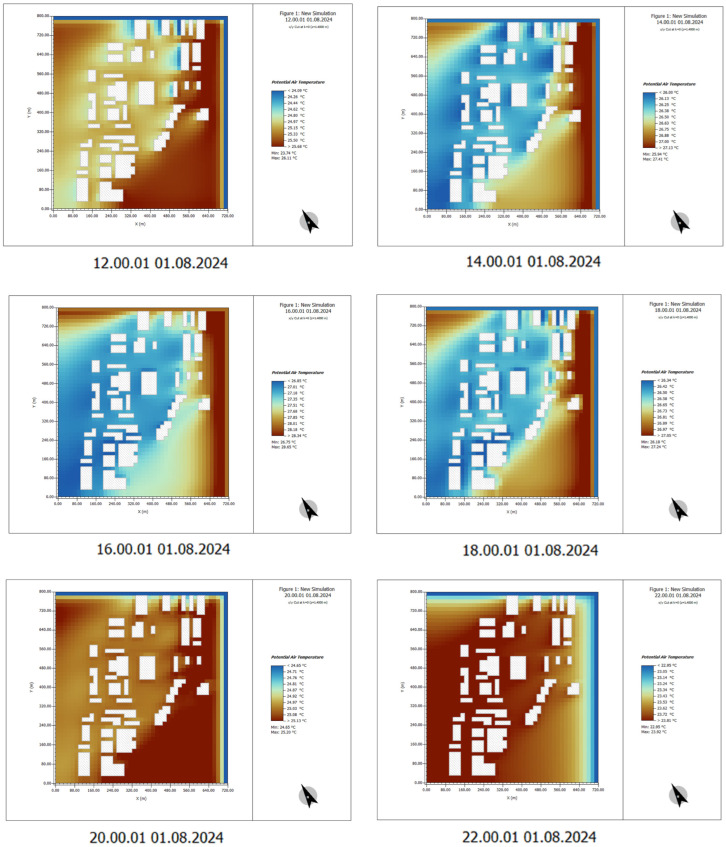
Potential temperature simulation results. Time: 12:00–22:00. (The white shape represents the building).

Between 16:00 and 18:00, temperatures gradually peak, with some areas reaching as high as 34–36 °C. The accumulation of heat on building surfaces and roads results in persistently high temperatures in these areas (Figure 10).

#### 4.2.2. Human Comfort Data Analysis

Human comfort is mainly determined by temperature, humidity, wind speed, and radiation. This study used PMV (predicted mean vote), which predicted the human body’s comfort level to the environment by comprehensively considering temperature, humidity, wind speed, radiation, and other factors. The value range ranged from −4 (extremely cold) to +4 (extremely hot), with 0 representing neutral and the most comfortable state [35]. Taking 1 August 2024 as the simulated measurement time, the PMV index data were obtained, and the results show the changes in human comfort. Because there is no shelter in these areas, the lack of greening and direct sunlight on the surface of the human body lead to the increase of radiant heat, making the perceived temperature of the human body higher than the air temperature. The ENVI-met-simulated PMV index showed that the PMV value on the south side of the building exceeded +3, indicating that the human body felt significant heat, and was exposed to direct sunlight, further aggravating the human body’s dry heat sensation. Visitors walking or staying in exposed areas are prone to dehydration or fatigue, especially children and older individuals, and are prone to feeling stuffy and uncomfortable in hot weather. Only at 14:00, when the building is shaded, does the temperature improve slightly, but the effect is minimal.

(1) Time: 00:00–10:00

From 00:00 to 08:00 in the morning, the PMV (predicted mean vote) values are generally low, indicating favorable thermal comfort, which suggests that the air temperature is relatively low and the environment is more comfortable. However, as the time progresses from 08:00 to 10:00, the PMV values begin to rise gradually, particularly in the areas surrounding the building. This indicates that the temperature is increasing, and the sensation of thermal discomfort starts to intensify.

**Figure 11:**
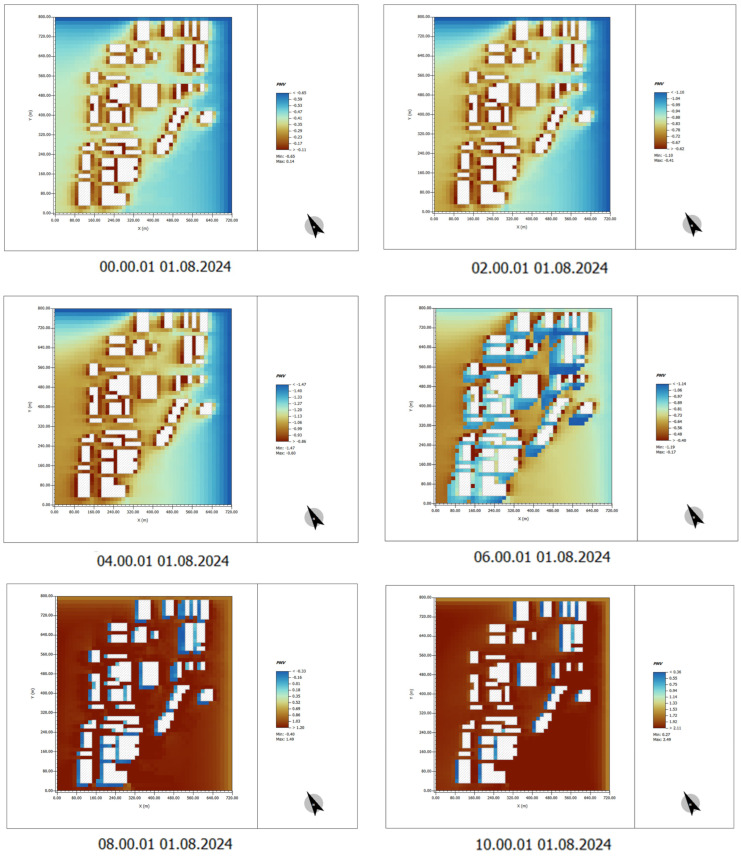
PMV indicator data results. Time: 0:00–10:00. (The white shape represents the building).

Particularly near the exposed surfaces of the building, the PMV values are significantly higher, suggesting that the temperature in these areas rises rapidly, leading to an increase in thermal discomfort (Figure 11).

(2) Time: 12:00–22:00

During the high-temperature period from 12:00 to 16:00, the PMV values reach their peak, especially in areas around buildings and outdoor spaces. This indicates a strong sensation of thermal discomfort in these regions. When solar radiation is intense, PMV values typically exceed 1.0, signifying a hot environment.

From 16:00 to 20:00, as the intensity of sunlight gradually weakens, the PMV values begin to decline, but they remain relatively high in certain areas, indicating that thermal comfort is still poor, particularly on building surfaces and open spaces.

**Figure 12:**
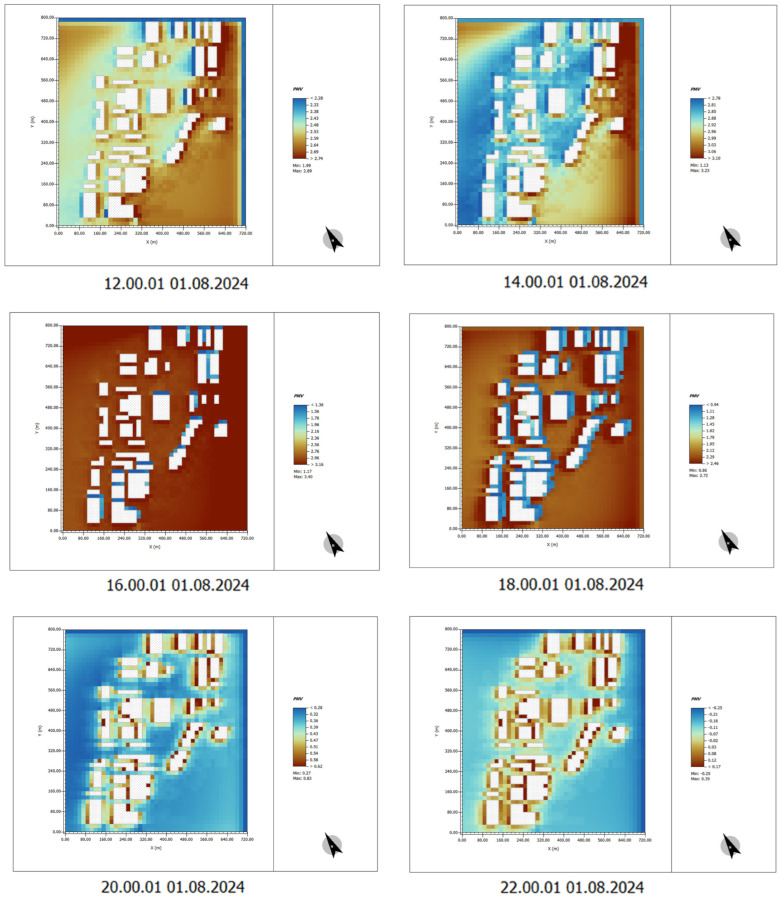
PMV indicator data results. Time: 12:00–22:00. (The white shape represents the building).

After 20:00, the PMV values tend to stabilize. Although the environment becomes somewhat cooler, the thermal mass retention in the buildings causes the PMV values in some areas to remain above the ideal level (Figure 12).

#### 4.2.3. Wind Speed Distribution Analysis

Areas with Low Wind Speed: The yellow and orange regions in the map represent areas with lower wind speeds, typically located in densely built-up areas or places with limited open space. In these areas, the airflow is restricted, leading to poor air circulation, which causes heat to accumulate and reduces thermal comfort.

Areas with High Wind Speed: The green and blue regions represent areas with higher wind speeds, usually found in more open spaces or between buildings. Although these areas have higher wind speeds, the overall distribution of wind speed is uneven, with many places experiencing lower wind speeds, which limits airflow.

Impact Analysis:

Wind Speed Reducing Comfort: In the absence of plant design, the lack of vegetation leads to a reduction in the guidance of airflow and cooling effects. This results in uneven wind speed distribution. In areas with lower wind speeds, air circulation is poor, making it difficult for temperatures to decrease effectively, thus exacerbating thermal discomfort, especially around buildings and areas without greenery.

**Figure 13:**
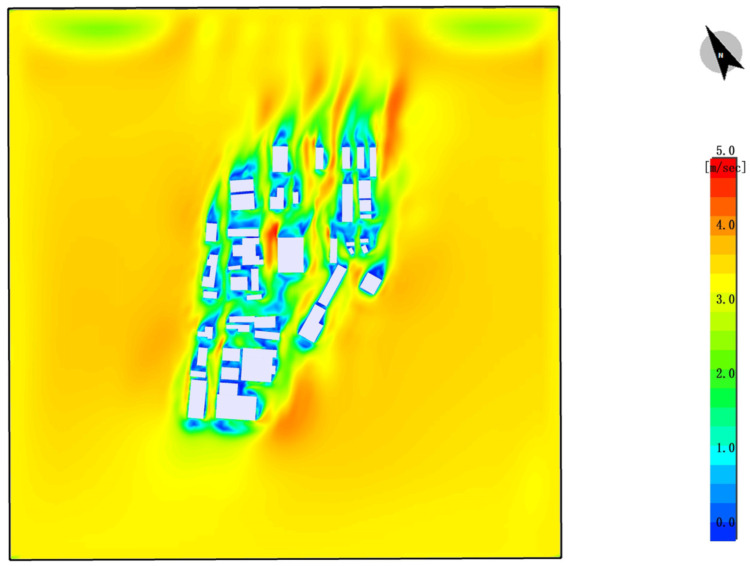
Wind speed distribution. (The white shape represents the building).

Intensified Urban Heat Island Effect: Uneven wind speed distribution can exacerbate the urban heat island effect. In areas without plants, there is often a lack of airflow, preventing heat from dissipating effectively, which further impacts comfort, particularly around buildings and near hard surfaces (Figure 13).

#### 4.2.4. UTCI Analysis

The UTCI (universal thermal climate index) thermal comfort distribution map demonstrates the thermal comfort conditions of the site in the absence of plant vegetation. The following characteristics can be observed from the color distribution:

High-Temperature Areas: The red and orange areas in the map indicate regions with higher temperatures. These areas are primarily concentrated around hard surfaces such as metal and concrete surfaces of buildings. These surfaces absorb and store significant amounts of solar heat, resulting in higher local temperatures.

Urban Heat Island Effect: In areas without plants, the temperature is notably higher, causing a strong urban heat island effect. In these high-temperature areas, human thermal comfort is lower, leading to discomfort, especially near exposed hard surfaces.

**Figure 14:**
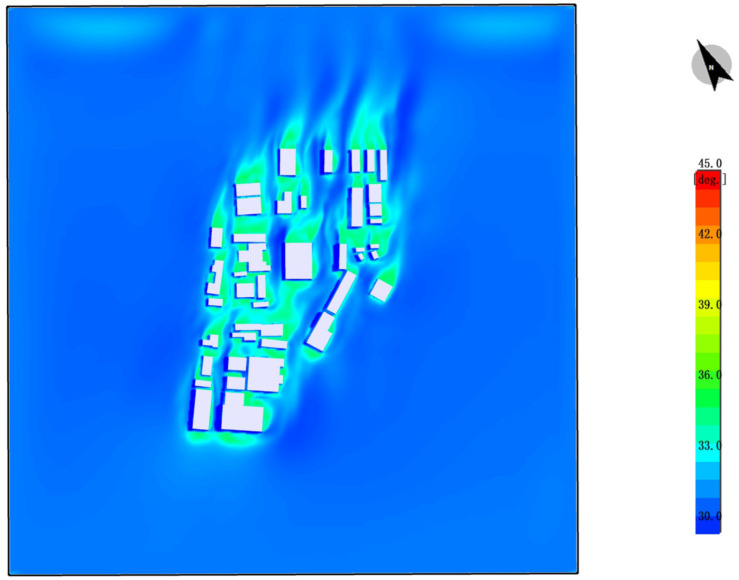
UTCI. (The white shape represents the building).

Insufficient Wind Speed: Due to the lack of vegetation or green spaces that typically guide airflow and provide cooling effects, air movement is obstructed. This prevents the temperature from effectively decreasing, further intensifying the discomfort caused by heat (Figure 14).

### 4.3. Plant Landscape Design

Through the above experimental results, it can be seen that the traditional hard urban infrastructure (such as concrete and metal structures) significantly intensifies the heat island effect, while the introduction of green infrastructure, such as plant landscape design, can adjust the temperature through natural means and reduce the high temperature effect in the region.

#### 4.3.1. Overall Layout

**Figure 15:**
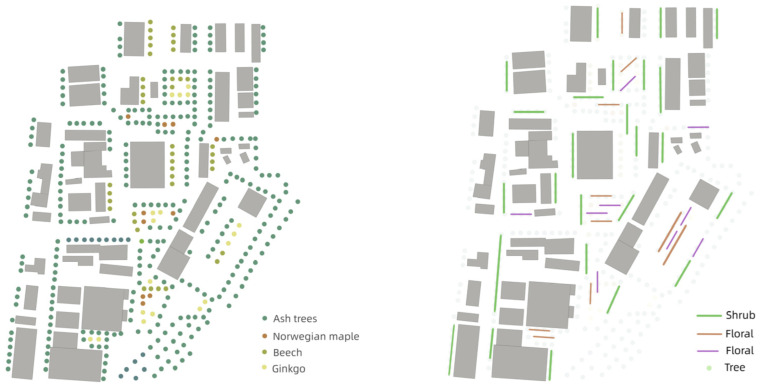
Plant landscape design plan.

Around the building, the design focuses on adding trees to provide effective shade and reduce the direct impact of solar radiation on the building surface and ground. The core of the whole plant design scheme is to balance the historical characteristics of the industrial site with modern ecological needs to create a functional and beautiful green public space, which not only meets the needs of environmental transformation, but also enhances the comfort and experience of visitors (Figure 15).

#### 4.3.2. Plant Species

(1) Design of tree layer

We selected European ash, European beech, Norway maple, and Ginkgo as the main street trees. European ash has a high environmental tolerance, making it resistant to temporary flooding, waterlogging, and drought conditions. As a result, European ash is often used as a street tree in urban areas [36,37]. European beech has excellent shade tolerance [38]. The high leaf density of Norway maple can effectively reduce wind speed and create a stable air environment [39]. Ginkgo, commonly used in landscape spaces, has an aesthetic appeal with its seasonal changes [40]. These trees also provide shading through their canopies, effectively reducing sunlight exposure on the ground and buildings, thereby minimizing the direct impact of solar radiation on building surfaces and the ground [41,42].

(2) Design of shrub layer

The shrub layer included Spiraea (Lilac), Elderberry (Elderberry), Spindle, and Rhododendron. Shrubs are often used around buildings and at the edges of open spaces to form natural transition zones while enhancing privacy. These shrubs not only visually enrich the landscape, but also provide additional shading to local areas through dense foliage and facilitate air flow in the microenvironment [43]. Shrubs also attract insects and birds, increasing ecological diversity and improving the ecological balance of the entire area. Flowering shrubs such as rhododendrons and cloves add colour to the landscape and attract visitors through their seasonal flowering.

(3) Design of flower layer

**Figure 16:**
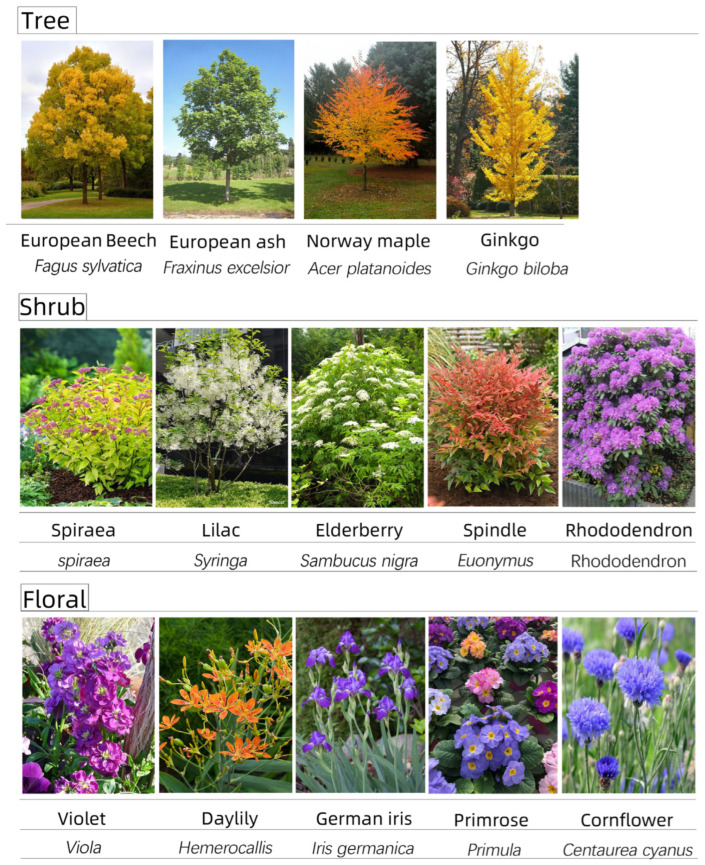
Plant species analysis.

The floral layer consisted of Violet, Daylily, German iris, Primrose, and Cornflower. These flowers add movement and vitality to the landscape through their rich colors and diverse forms, attract more insects and small animals, and provide a unique visual enjoyment for visitors. Especially in the walkways and rest areas, the floral design enhances the appeal of the space, adds a sense of seasonal change, and enhances the visitor experience (Figure 16).

#### 4.3.3. Vegetation Layers

**Figure 17:**
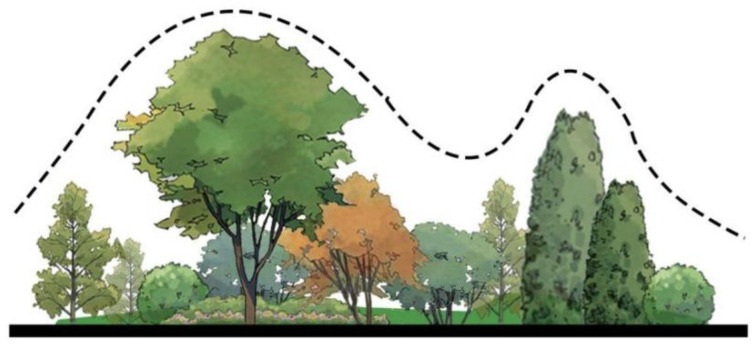
Plant hierarchy analysis.

In the elevation drawing, local native tree species are used to rationally arrange plants of different heights and forms to form a beautiful plant community. In addition, the different canopy shapes of plants also constitute the scattered canopy lines. These landscape plant elements not only visually beautify the site space of industrial sites, but also make the environment here full of vitality (Figure 17).

The tallest plants belong to the tree layer, which usually reaches a higher height and is the top layer of the entire plant community. The tall tree canopy forms the main visual framework in the landscape, providing a sense of vertical space. Their diverse tree species and seasonal variations (such as the autumn golden leaves of Ginkgo biloba) add rich visual layers [44].

The shrub layer is the middle layer in the elevation and usually contains medium height plants (such as rhododendrons, cloves, etc.). They fill the space between trees and ground cover and enrich the three-dimensional sense of the entire landscape. They can act as a natural transition to the space, connecting the visual effects of trees and ground cover. The ground floor is at the bottom of the elevation and consists mainly of low-growing plants, such as herbs and small flowers (violets, irises, etc.). In terms of visual effects, ground cover plants add vitality to the landscape through their diverse colors and flowering periods, and decorate the entire landscape at the bottom. The harmonious symbiosis of these different levels of plants creates an ecological green infrastructure for the Henrichshutte industrial site, providing the necessary conditions for ecological restoration and landscaping in the region.

#### 4.3.4. Comparison of Plant Landscape

**Figure 18:**
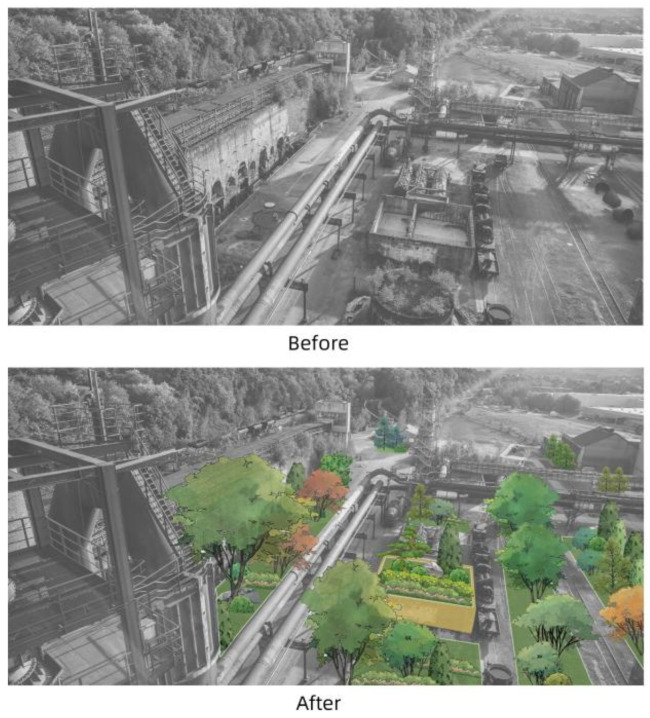
Before-and-after comparison of plant landscape design.

The picture shows the comparative effect of industrial sites before and after greening. The non-green space is dominated by abandoned industrial facilities and hard paving. After plant design, the space of the site has bright colors and a sense of layers. The design of trees, shrubs, ground covers, and other multi-level plants not only improves the visual experience, but also enhances the ecological function of the site (Figure 18).

### 4.4. Analysis of Temperature Change After Plant Landscape Design

To address the high-temperature issues in the Henrichshütte steel plant and the surrounding factory spaces, we designed a multi-layered ecological vegetation community based on the concept of GI to mitigate the microclimate and comfort issues of the site. From the simulation results of ENVI-met, it is evident that the integration of plant landscapes into the previously exposed buildings and open areas significantly reduces the potential temperature.

(1) Time: 00:00–10:00

In the images with plant design, the overall temperature of the area is lower, particularly in the regions surrounding the building. Compared to the images without plant design, the canopy and green spaces effectively reduce the surface temperature of the ground and building facades, thereby mitigating heat accumulation.

**Figure 19:**
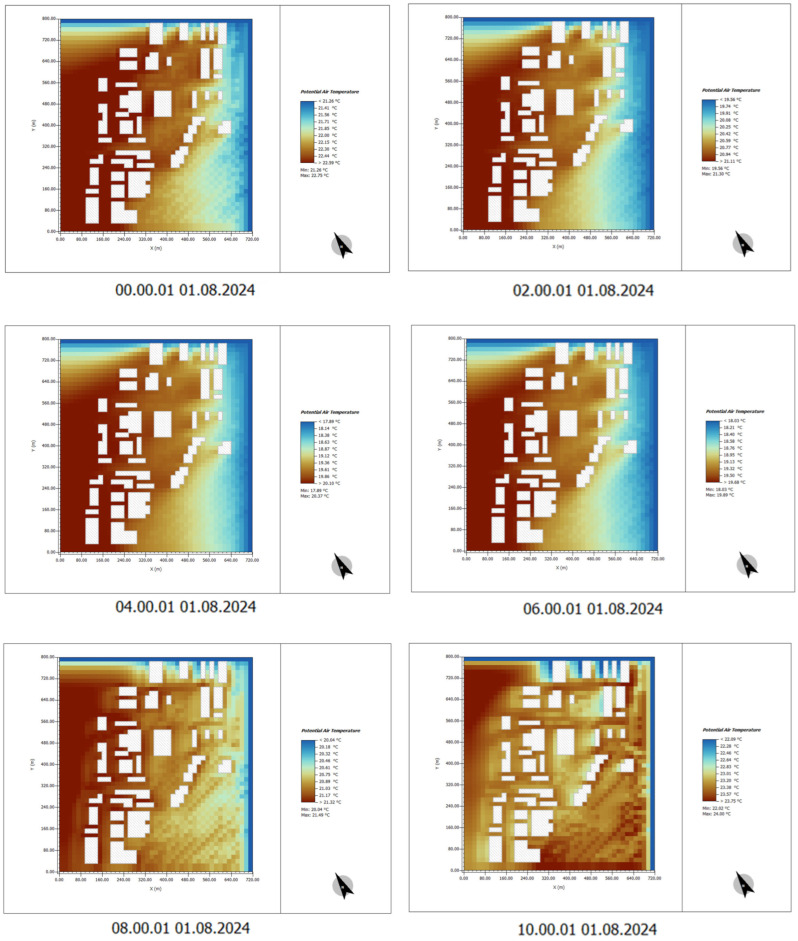
Potential temperature simulation results. Time: 00:00–10:00 (After plant landscape design).

For instance, at 06:00 and 08:00, the temperature in green spaces and planted areas is relatively lower, while the exposed building surfaces remain warmer, though this effect is less pronounced compared to areas without plant design (Figure 19).

(2) Time: 12:00–22:00

From 12:00 to 22:00, the effects of plant design become more evident. During periods of intense sunlight, the tree canopies effectively provide shading to the ground and building surfaces, significantly lowering the temperature, particularly during the midday and afternoon high-temperature periods.

For example, at 12:00 and 14:00, the temperature of building surfaces shows a noticeable decrease, especially in areas with vegetation coverage. In contrast, in the absence of plant design, the temperature of building facades and open spaces remains higher, exacerbating the urban heat island effect.

**Figure 20:**
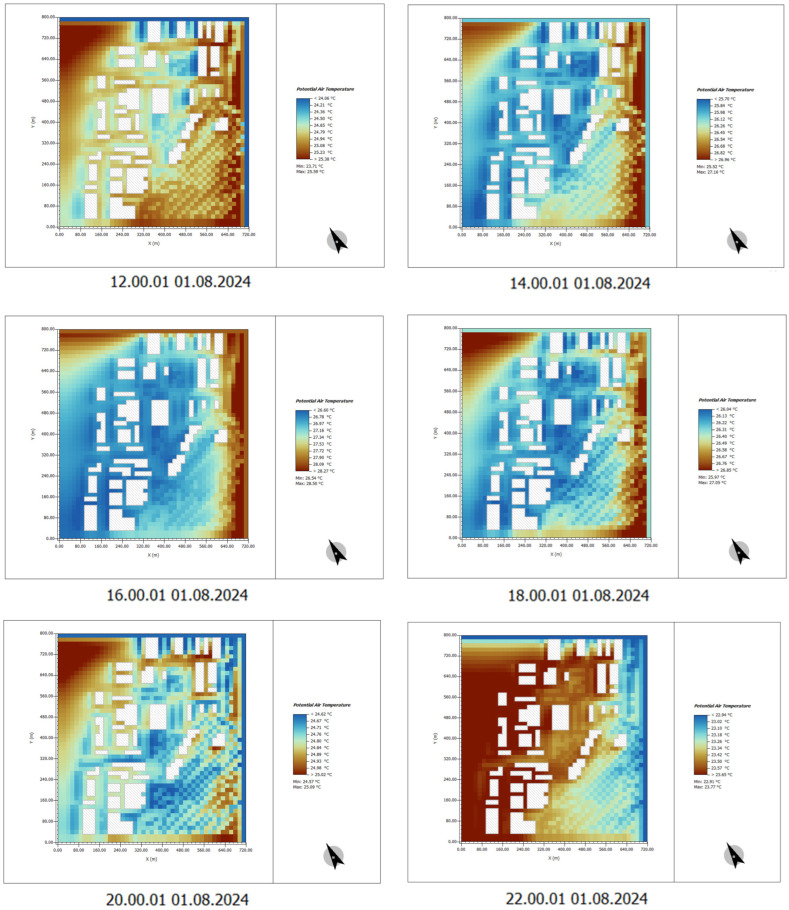
Potential temperature simulation results. Time: 12:00–22:00 (After plant landscape design).

In the evening, plant design continues to help maintain lower temperatures on the ground and building surfaces, although the overall temperature has begun to decrease (Figure 20).

### 4.5. Analysis of Human Comfort After Plant Landscape Design

By analyzing the predicted mean vote (PMV) simulation diagram of human comfort after greening, the effect of plant design on improving human comfort can be intuitively analyzed.

(1) Time: 00:00–10:00

In the images with plant design, the overall PMV values are significantly lower compared to those without plant design, especially in areas surrounding buildings and open spaces. Vegetation and green spaces provide better shading, lowering temperatures and improving thermal comfort.

**Figure 21:**
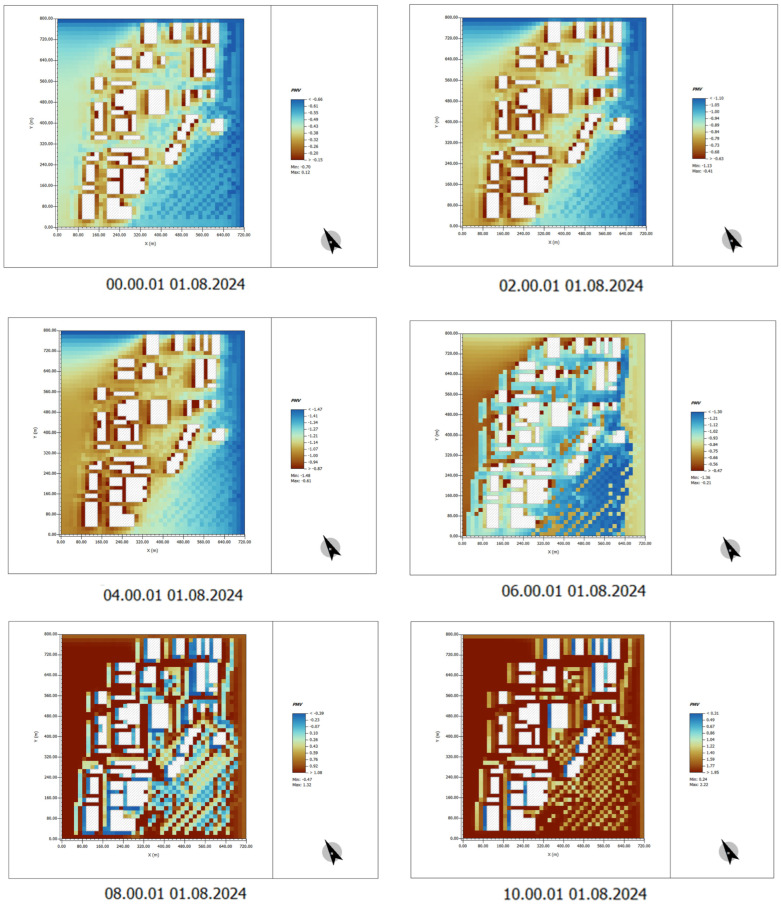
PMV indicator data results. Time: 00:00–10:00 (After plant landscape design).

Between 00:00 and 06:00, the PMV values are generally low, indicating favorable thermal comfort and relatively pleasant air temperatures. As time progresses to 08:00–10:00, although temperatures begin to rise, the areas with plant design still maintain lower PMV values, demonstrating the positive role of plants in temperature reduction and enhancing thermal comfort (Figure 21).

(2) Time: 12:00–22:00

During the high-temperature period from 12:00 to 16:00, the PMV values in the areas surrounding buildings and open spaces are notably lower compared to those without plant design. The shading effect of trees significantly mitigates the impact of solar radiation on the ground and building surfaces, greatly reducing thermal discomfort in these areas.

From 16:00 to 20:00, although temperatures decline, the PMV values in areas with plant design remain relatively low, indicating that thermal comfort is better maintained in these areas.

**Figure 22:**
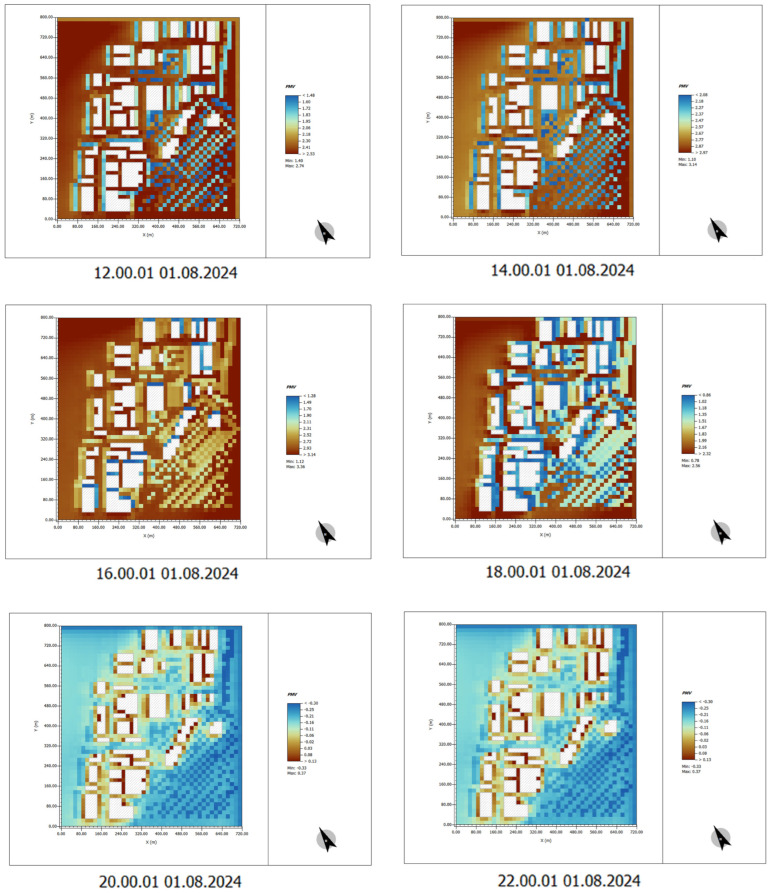
PMV indicator data results. Time: 12:00–22:00 (After plant landscape design).

After 20:00, the PMV values stabilize. Even in building and open areas, the plant design continues to effectively maintain lower PMV values, thereby enhancing overall thermal comfort (Figure 22).

### 4.6. Wind Speed Distribution

**Figure 23:**
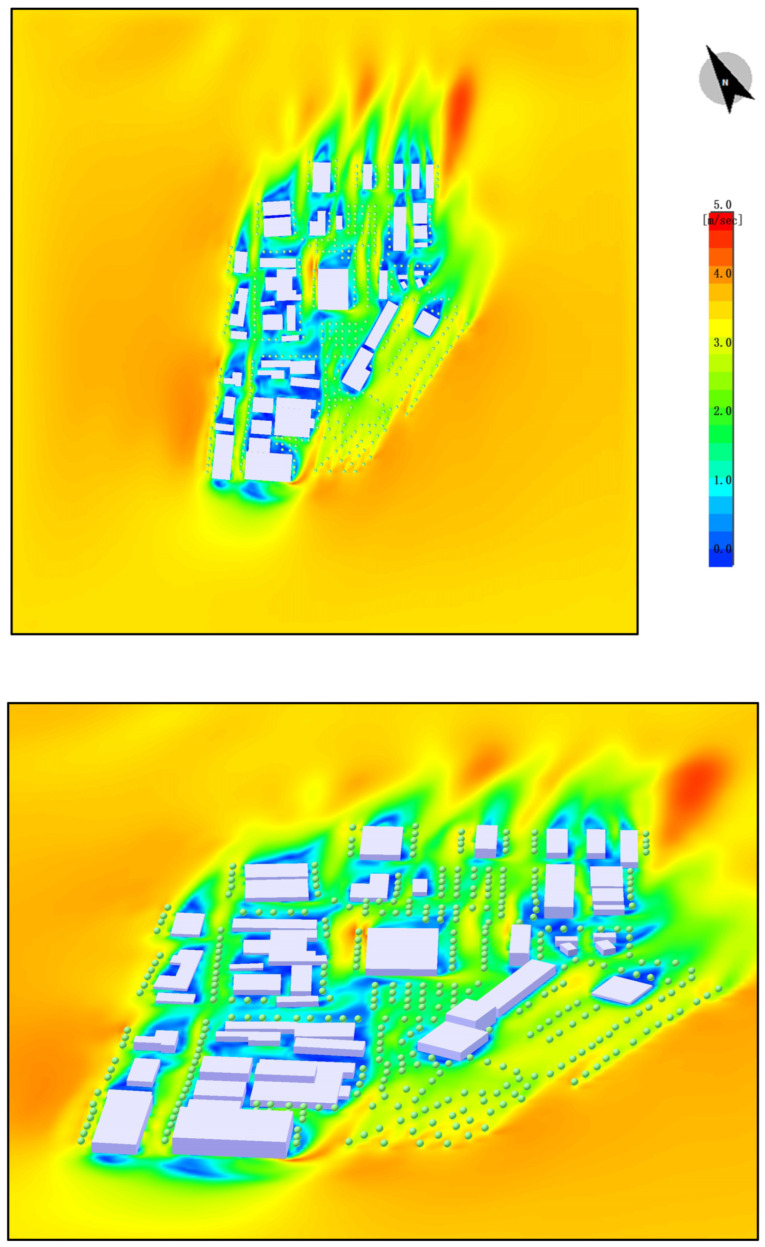
Wind speed distribution (After plant landscape design).

In areas with plant design, the presence of vegetation and green spaces improves the distribution of wind speed, leading to a more uniform wind flow, especially in green spaces and tree-planted areas where wind speeds are higher. Vegetation effectively alters wind flow patterns, reducing the obstruction caused by buildings and enhancing local airflow. Plant design enhances wind speed in localized areas, improving air circulation and ventilation. This contributes to reducing the urban heat island effect and enhances thermal comfort (Figure 23).

The image displays the UTCI data results after the introduction of plant vegetation. By observing the color distribution in the map, the following conclusions can be drawn:

Temperature Reduction: The areas with higher temperatures have become fewer, as the red and orange regions shrink, with some areas turning green and blue, indicating a significant decrease in temperature. The introduction of plants effectively reduces the surrounding environment’s temperature through processes such as transpiration, shading, and increasing air humidity.

Improved Thermal Comfort: The decrease in temperature results in a significant improvement in human thermal comfort, particularly in areas covered with plants. By absorbing and releasing moisture, and providing shade, plants reduce the impact of solar radiation on the ground, effectively mitigating the urban heat island effect.

**Figure 24:**
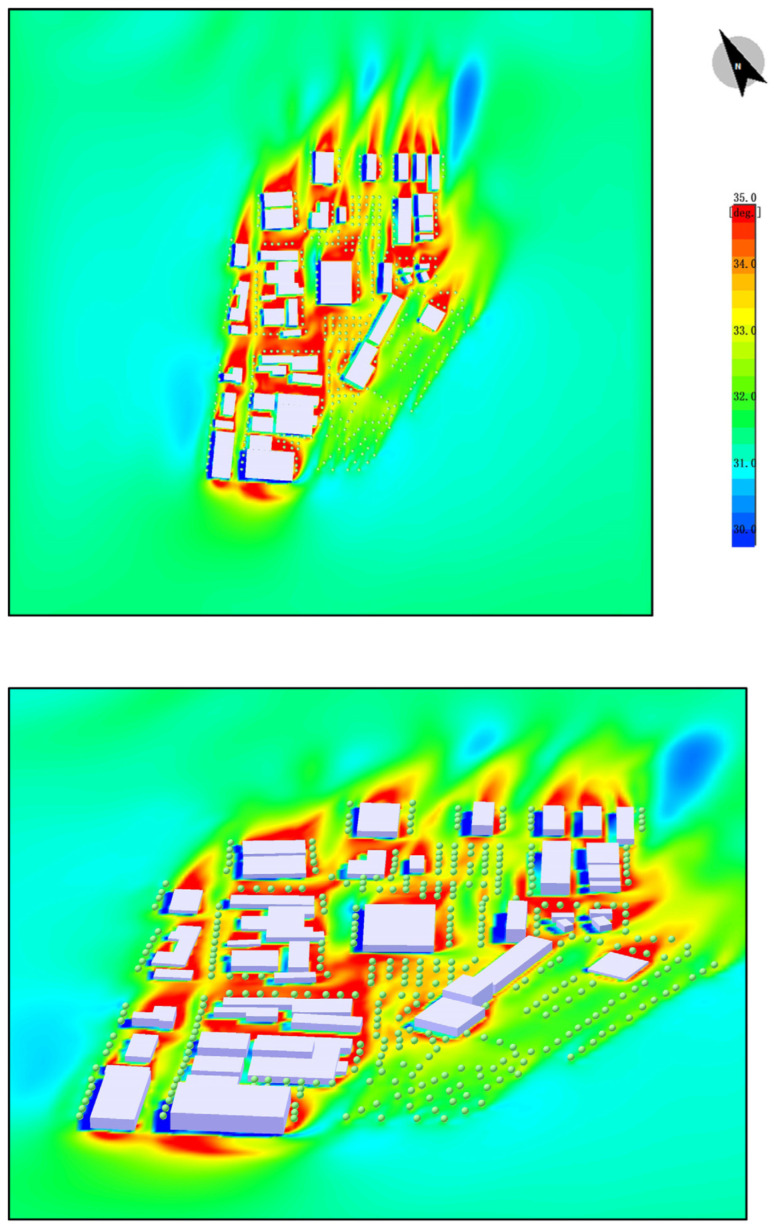
UTCI (After plant landscape design).

Improved Wind Environment: The introduction of plants not only helps lower the temperature but also improves the surrounding wind environment. Plants and green spaces can guide airflow and enhance ventilation, making air circulation smoother. This further lowers the temperature in surrounding areas and enhances thermal comfort (Figure 24).

## 5. Discussion

This study validates the effectiveness of green infrastructure (GI) concepts in improving the microclimate and comfort conditions of urban industrial heritage sites through a simulated analysis of the Henrichshütte steel plant and its surrounding factory areas. The results demonstrate that landscape design significantly enhances environmental conditions, particularly in terms of temperature regulation, wind speed distribution, and thermal comfort.

On one hand, plant design plays a critical role in temperature regulation. Particularly in summer, the accumulation of heat in deteriorating steel industrial equipment and concrete buildings causes local temperature increases, resulting in discomfort due to heat radiation. Simulation results show that vegetation design, particularly in ecological plant communities, significantly reduces the surface temperatures of both ground and buildings. Vegetation provides shading, increases soil moisture, and reduces heat accumulation, especially during the midday to evening periods, where the cooling effect is most pronounced. This cooling effect effectively mitigates the urban heat island phenomenon and creates a more comfortable environment for people.

On the other hand, plant landscape design also plays an important role in improving wind conditions. By comparing simulation results, it was found that wind speed distribution in greened areas was more uniform, and air circulation was optimized. Plants enhance air flow and optimize wind speed distribution, improving microclimatic conditions and overall ventilation and comfort. This is particularly important for enhancing thermal comfort, as improved air circulation in hot summer months can effectively reduce the thermal load on the human body.

In addition, the transpiration effect of plants has a significant impact on increasing air humidity and regulating local temperature. Plants regulate air humidity through water evaporation and produce a cooling effect on temperature, further enhancing spatial comfort. Especially during high summer temperatures, the integrated regulation of temperature and humidity by plants creates a more pleasant environment.

Furthermore, this study confirms the effectiveness of multi-layered plant community design. Compared to single-layer plant communities, multi-layered plant groups provide more complex microclimate regulation effects. Through the complementary functions of different plant species, temperature, humidity, and air flow distribution are improved. The results show that well-designed multi-layered plant communities not only effectively regulate environmental temperature but also optimize air circulation, further enhancing the ecological and comfort qualities of the area.

## 6. Conclusions

This study systematically analyzes the impact of plant landscape design guided by the concept of GI on the green space environment of urban industrial sites. Through simulation experiments, the study found that plant landscape design significantly improved thermal comfort and wind environment in industrial site green spaces during the summer, particularly with the design of multi-layered plant ecological communities, which notably optimized thermal comfort and wind speed distribution. Specifically, plant design regulated environmental temperatures, increased wind speed uniformity, and provided comfortable shading, which not only alleviated the urban heat island effect but also improved thermal comfort and ventilation conditions for the residents.

This finding is consistent with previous studies, further validating the effectiveness of GI in landscape spaces and extending its practical significance in the transformation of industrial sites. By comparing the simulation results of industrial site spaces and surrounding factory buildings under different conditions, this study reveals the significant impact of multi-layered plant communities on thermal comfort and wind environment, demonstrating the potential of plant landscape design for environmental regulation.

In addition, the study highlights the positive contribution of plant landscape design to ecological restoration. As an effective ecological restoration tool, Green Infrastructure plays a crucial role in the spatial transformation of industrial sites. By optimizing environmental conditions, plant landscapes not only enhance spatial comfort but also provide more pleasant leisure and activity spaces for citizens, promoting the sustainable development of urban spaces.

Although the experimental site sample size in this study is relatively small, and the research focuses mainly on specific types of industrial sites, certain limitations exist. Nevertheless, these findings provide valuable theoretical support and practical guidance for the regeneration of industrial sites and urban environmental design in the future. To further validate the generalizability of the results, future research should expand to include different types of industrial sites and broader regions, increasing the diversity of experimental samples and improving the reliability of the findings.

## Data Availability

The original contributions presented in the study are included in the article; further inquiries can be directed to the corresponding author.
